# Temporary interruption of baricitinib: characterization of interruptions and effect on clinical outcomes in patients with rheumatoid arthritis

**DOI:** 10.1186/s13075-020-02199-8

**Published:** 2020-05-15

**Authors:** Paul Emery, Yoshiya Tanaka, Tracy Cardillo, Douglas Schlichting, Terence Rooney, Scott Beattie, Cameron Helt, Josef S. Smolen

**Affiliations:** 1 Leeds Muscoloskeletal Biomedical Research Centre/Chapel Allerton Hospital, Chapeltown Rd, Leeds, LS7 4SA UK; 2 The First Department of Internal Medicine, School of Medicine, University of Occupational and Environmental Health, Kitakyushu, Japan; 3 Eli Lilly and Company, Indianapolis, IN USA; 4 Division of Rheumatology, Department of Medicine 3, Medical University of Vienna, Vienna, Austria

**Keywords:** Rheumatoid arthritis, DMARDs (biologics), JAK inhibitors, Drug interruption

## Abstract

**Background:**

In clinical practice, temporary interruption of rheumatoid arthritis (RA) therapy is common for various reasons including side effects, non-compliance, or necessity for surgery. To characterize temporary interruptions of baricitinib and placebo-matched tablets in phase 3 studies of patients with moderate-to-severe rheumatoid arthritis (RA) and describe their impact on efficacy and safety.

**Methods:**

During 4 baricitinib phase 3 studies, investigators documented timing, reason, and duration of investigator-initiated temporary interruptions of study drug. In 2 studies, patients recorded RA symptoms in daily diaries for 12 weeks. Post hoc analyses investigated changes in symptom scores during interruptions and resumption of treatment. Interruptions were evaluated for reoccurrence of adverse events or laboratory abnormalities after retreatment.

**Results:**

Across the placebo-controlled studies, interruptions occurred in larger proportions of baricitinib- (2 mg, 18%; 4 mg, 18%) vs placebo-treated (9%) patients in only one study (bDMARD-inadequate responder patients, RA-BEACON). In the active comparator-controlled studies, the lowest rates of interruption were in the baricitinib monotherapy arm (9%) of RA-BEGIN (vs methotrexate monotherapy or combination therapy), and proportions were similar for baricitinib (10%) and adalimumab (9%) in RA-BEAM. Adverse events were the most common reason for interruption, but their reoccurrence after drug restart was infrequent. Most interruptions lasted ≤ 2 weeks. Daily diaries indicated modest symptom increases during interruption with return to pre-interruption levels or better after resumption. Interruptions had no impact on long-term efficacy outcomes.

**Conclusions:**

Consistent with its pharmacologic properties, brief interruptions of baricitinib during phase 3 studies were associated with minor increases in RA symptoms that resolved following retreatment. This analysis provides useful information for clinicians, as temporary interruption of antirheumatic therapy is common in the care of patients with RA.

**Trial registration:**

ClinicalTrials.gov; NCT01710358, NCT01711359, NCT01721057, NCT01721044

## Background

Temporary interruption of rheumatoid arthritis (RA) therapy is common in clinical practice for various reasons including side effects, non-compliance, or necessity for surgery. Short half-life and low immunogenicity may therefore be useful attributes for disease-modifying antirheumatic drugs (DMARDs). Few studies examine what happens when DMARDs are temporarily interrupted and then restarted despite concerns of symptoms worsening or immunogenicity increasing after the stoppage. If disease activity deteriorates, there is also the question of whether it will improve once the drug is started again.

Baricitinib is an oral, selective inhibitor of Janus kinase (JAK)1 and JAK2, which belong to a family of protein tyrosine kinases that mediate signal transduction for a variety of cytokines involved in inflammatory conditions, including RA [1, 2]. As a small molecule with a short half-life (approximately 12 h in RA patients) [3], baricitinib may offer advantages over injectable biologic therapies with respect to ease and speed of withdrawal and re-initiation.

Baricitinib improved signs and symptoms of RA in 4 phase 3, placebo- and active-controlled studies in patients with active RA who were naïve to conventional synthetic disease-modifying antirheumatic drugs (csDMARDs) (RA-BEGIN) [4] or had an inadequate response to previous treatment with methotrexate (MTX) (RA-BEAM) [5], csDMARDs (RA-BUILD) [6], or biologic DMARDs (RA-BEACON) [7]. The objectives of this analysis were to characterize temporary interruptions of baricitinib (and the placebo tablets in the active comparator arms) during these studies and examine the impact of interruptions and drug retreatment on efficacy and safety outcomes.

## Methods

### Study design and patients

Data were included from 4 phase 3 randomized clinical studies. The study design and patient inclusion/exclusion criteria for each study have been described previously [4–7]. Briefly, 684 patients in RA-BUILD (NCT01721057) and 527 patients in RA-BEACON (NCT01721044) with active RA were randomized 1:1:1 to receive placebo or 2- or 4-mg baricitinib once daily (QD); 1305 patients in RA-BEAM (NCT01710358) were randomized 3:3:2 to receive placebo QD or 4-mg baricitinib QD or a subcutaneous injection of adalimumab every 2 weeks; and 588 patients in RA-BEGIN (NCT01711359) were randomized 4:3:4 to receive oral MTX every week (QW) or 4-mg baricitinib QD or 4-mg baricitinib QD plus MTX QW. Beginning at week 16 (week 24 in RA-BEGIN) in each of the studies, non-responders, defined as patients with a lack of improvement of at least 20% in both tender joint count and swollen joint count compared to baseline, received rescue treatment as outlined in Additional file 1: Table S1. All patients completing these 4 phase 3 studies were eligible to enter the long-term extension study, RA-BEYOND.

Patients who were not receiving glucocorticoids prior to randomization were not permitted to initiate glucocorticoid therapy during the study, including intra-muscular or intra-articular glucocorticoids. After rescue, new glucocorticoids or increases in doses of ongoing concomitant glucocorticoids were permitted. Topical, intranasal, intra-ocular, and inhaled glucocorticoids were permitted.

The primary endpoint in the studies was the American College of Rheumatology 20% (ACR20) response rate at week 12 (week 24 in RA-BEGIN). In RA-BEAM and RA-BUILD (the two studies in which patients had an inadequate response to csDMARDs), patients recorded daily symptoms in an electronic diary from the first day of treatment through week 12, referred to as diary patient-reported outcomes (PROs). Diary entries included duration of morning joint stiffness (MJS) and numeric rating scales (NRS) for MJS severity, worst tiredness, and worst joint pain. Scores for the NRS ranged from 0 to 10, with 10 being the worst level [8]. Additional details regarding the study designs can be found in Additional file 1: Table S1.

Each study was conducted in accordance with the principles of the Declaration of Helsinki and Good Clinical Practice Guidelines and approved by each center’s institutional review board or ethics committee. All patients provided written informed consent. The studies were designed by the sponsors, Eli Lilly and Company and Incyte Corporation, with input from an academic advisory board in which non-Lilly authors of this manuscript participated. All authors participated in the preparation and review of this manuscript and approved the final version.

### Characteristics of interruptions

During each study, temporary interruptions of study drug initiated by the investigators were required to be documented in an electronic case report form, providing the time of the last dose, the reason for the interruption, and the duration of the interruption. On the form, investigators could choose one of 4 reasons for the interruption: “adverse event (AE),” “abnormal laboratory values,” “suspected pregnancy,” or “investigator decision.” If investigators selected “AE” as the reason for the interruption, they were immediately prompted to link a specific AE to the interruption in the electronic case report form. Investigators might have listed an abnormal laboratory value as an “AE,” rather than selecting the separate option of “abnormal laboratory value” as the reason for the interruption. In these instances, we reviewed the abnormal laboratory values individually to gain a more complete understanding of laboratory abnormalities that led to interruptions. If “abnormal laboratory value” was selected as the reason for interruption, a query was sent to the investigator for specific information regarding the type of laboratory test and abnormal finding.

Temporary interruption was defined as a temporary withholding of study drug that was followed by the resumption of study drug at a later time point. Interruptions were based on the daily tablet baricitinib study drug, including non-baricitinib groups, which represented interruptions of the matching placebo for baricitinib. In some instances, the study drug was interrupted with the intent of restarting, but instead led to a permanent discontinuation of treatment, which will be referred to as “initiated interruptions.” Patients who had their daily tablet of baricitinib or matching baricitinib placebo temporarily interrupted were compared across their originally assigned treatment groups for trends in the frequency, duration, and reason for baricitinib interruptions within each study. For interruptions among all baricitinib-treated patients, including those who were originally randomized to placebo or active-control, but were rescued or switched to baricitinib during the studies, we provide a detailed summary of the reasons for the interruptions.

### Impact of interruptions on efficacy

Post hoc analyses to assess the impact of interruptions on efficacy outcomes in the short- and long-term were conducted in patients who were csDMARD-inadequate responders (IR) and MTX-IR using pooled data of patients randomized to placebo and baricitinib in RA-BEAM and RA-BUILD. First, the effect on longer-term efficacy outcomes was assessed by the percentage of patients with ACR20 and ACR50 response and with Disease Activity Score using 28 joints based on C-reactive protein (DAS28-CRP) ≤ 3.2 at week 24. Responses were compared between patients with and without interruptions of any duration during the first 24 weeks or up to rescue.

Second, although standard RA assessments of efficacy response and disease activity measures, with their associated acute phase reactants, were collected only at scheduled visits approximately every 4 weeks, the effect on short-term efficacy outcomes was assessed through the symptoms recorded by daily diary during the first 12 weeks of RA-BEAM and RA-BUILD. These diary scores were examined at time landmarks in patients who were randomized at least 7 days prior to interruption, had an interruption that lasted at least 3 days, and were then retreated with study drug. Symptoms were evaluated at the following milestones:

1. Study drug initiation (average of values obtained within the first 3 days following randomization)
2. Pre-interruption (average of up to 3 most recent values obtained in the 7 days prior to interruption)
3. Last scores during the interruption (average of 3 most recent values during the last 7 days of the interruption)
4. Post-interruption (average of last 3 available values obtained following interruption and prior to any subsequent interruption or week 12 study visit).

These 4-point profiles were compared between patients treated with and interrupting baricitinib (2 mg and 4 mg doses combined) and patients treated with and interrupting matching placebo.

To further explore the short-term loss of response and increase of symptoms during interruptions, an additional analysis was conducted of the 4 daily diary measures for the first temporary interruption per patient during the first 12 weeks when these measures were collected. For each measure, patients were classified into 1 of the following categories:

1. Patients who reported no worsening (possibly even improvement) of symptoms during the interruption.
2. Patients who reported only minimal worsening of symptoms during the interruption, defined as an increase of < 3 units of the NRS or ≤ 30 min of MJS duration. An increase of < 3 units in the NRS was chosen as representative of a change within severity category based on prior use of 3- to 4-point spans between severity categories of mild (0–3), moderate (4–6), and severe (7–10) for MJS [9]. An increase of 30 min of MJS duration was chosen as representative of a change within severity category based on prior use of 30-min spans between severity categories of mild (1–30 min), moderate (31–60 min), and severe (> 60 min) [10].
3. Patients who temporarily reported greater than minimal worsening of symptoms during the interruption, defined as an increase of 3+ units of the NRS or 31+ minutes of MJS duration, but whose last reported symptom scores during the interruption were improvements, not worsening, or minimal worsening.
4. Patients who reported greater than minimal worsening of symptoms during the interruption that did not resolve prior to resumption of treatment. For patients in this last category, the time for their symptoms to increase beyond minimal worsening was categorized as occurring during the first week, during the second week, or beyond the second week of the interruption. For MJS duration, these timings were considered for the first increase of symptoms by ≥ 61 min.

Because the numbers of patients for each of these efficacy assessments varied depending on efficacy and daily diary data available, the flow chart in Additional file 2: Table S2 outlines and explains the sample sizes for each assessment.

### Impact of interruptions on safety

Patient-level data were assessed to provide details on AEs and specific laboratory values that led to temporary interruptions. Reoccurrences of AEs and laboratory findings that led to a subsequent interruption after study drug was resumed were assessed via examination of patient-level data. For reoccurrence of AEs, matching preferred terms using the Medical Dictionary for Regulatory Activities (MedDRA) with a date subsequent to the date of drug resumption were identified in the database. For abnormal laboratory findings, a manual review of investigator-specified laboratory values was conducted to determine if abnormalities with these same analytes reoccurred once baricitinib was resumed. For a reoccurrence of the abnormal laboratory value to be considered, it had to be at least as high (or low) as the value that led to the initial interruption.

To assess whether initial tolerability effects persisted upon reinitiation of baricitinib, AEs reported during the first 4 weeks of treatment with baricitinib, from pooled data across all 4 studies, were compared to AEs reported in the 4 weeks after resuming baricitinib following a temporary interruption for the purpose of exploring whether the frequency and nature of the AEs were similar.

### Statistical analyses

Descriptive statistics are presented for the overall summary of interruptions, including reasons for and duration of interruptions and for efficacy and safety effects related to interruptions. No formal hypothesis tests were conducted to compare randomized treatment groups in terms of the number or nature of interruptions nor to compare specified groups with respect to the exploratory post hoc short-term and long-term efficacy analyses.

## Results

### Characteristics of interruptions

**Fig. 1:**
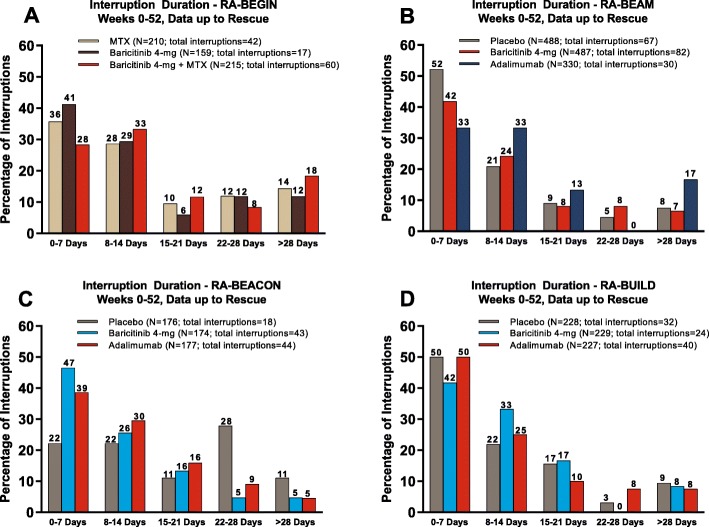
Duration of interruptions in the phase 3 studies RA-BEGIN (**a**), RA-BEAM (**b**), RA-BUILD (**c**), and RA-BEACON (**d**)^a,b^. ^a^Interruptions are based on daily tablet baricitinib study drug, including in non-baricitinib groups, which represent interruptions of the matching placebo for baricitinib. ^b^Temporary interruption is defined as a temporary withholding of study drug that is followed by resumption of study drug during the study. ^c^Percentage of interruptions. MTX, methotrexate

Temporary interruptions of baricitinib or matching placebo occurred in 8.5 to 18.1% of patients across treatment groups through week 24 and in up to 22.3% through week 52 (Table 1). During the 4 studies, there were 640 initiated interruptions of baricitinib or matching placebo across all treatment groups; in 84% of these cases (*n* = 536, range of 76–92%), the patient was able to restart study drug, thus defining these as temporary interruptions (Table 1). For patients on baricitinib during the placebo-controlled period of the studies (or the MTX-controlled period in RA-BEGIN), there were 343 initiated interruptions with 290 (85%) leading to reinitiation of baricitinib. In 2 of the 3 placebo-controlled studies (RA-BEAM and RA-BUILD), similar proportions of patients treated with placebo (11.1% and 12.7% in RA-BEAM and RA-BUILD, respectively) and 4-mg baricitinib (10.3% and 15.0%, in RA-BEAM and RA-BUILD, respectively) had interruptions, while the proportion of patients treated with 2-mg baricitinib (9.2%) in RA-BUILD with an interruption was slightly lower than the other two treatment groups. In RA-BEACON, there was a higher proportion of patients with interruptions with 4-mg baricitinib (18.1%) and 2-mg baricitinib (17.8%) than with placebo (8.5%) (Table 1). In the MTX active comparator-controlled study (RA-BEGIN), the proportion of patients with interruptions of baricitinib or matching placebo was lowest in the 4-mg baricitinib treatment group (Table 1). The time from the first dose of study treatment to interruption was longer in RA-BEGIN than in the other studies. In RA-BEAM, the duration of interruptions of baricitinib and placebo-matched tablets was shorter for the baricitinib treatment group compared to the adalimumab treatment group (Table 1, Fig. 1), although no inferential testing was performed.

**Table 1:** Summary of interruptions and temporary interruptions during baricitinib phase 3 studies in RA

	RA-BEGIN (0–52 weeks)^a^			RA-BEAM^a^					RA-BUILD (0–24 weeks)^a^			RA-BEACON (0–24 weeks)^a^		
				0–24 weeks			0–52 weeks^b^							
	MTX (*N* = 210)	BARI 4 mg (*N* = 159)	MTX+ BARI 4 mg (*N* = 215)	PBO (*N* = 488)	BARI 4 mg (*N* = 487)	ADA (*N* = 330)	BARI 4 mg (*N* = 487)	ADA (*N* = 330)	PBO (*N* = 228)	BARI 2 mg (*N* = 229)	BARI 4 mg (*N* = 227)	PBO (*N* = 176)	BARI 2 mg (*N* = 174)	BARI 4 mg (*N* = 177)
Initiated interruptions (*n*)	46	21	66	80	69	36	119	58	38	33	48	25	54	52
Study drug ever restarted	42 (91.3)	18 (85.7)	60 (90.9)	67 (83.8)	62 (89.9)	30 (83.3)	106 (89.1)	47 (81.0)	33 (86.8)	25 (75.8)	41 (85.4)	21 (84.0)	47 (87.0)	48 (92.3)
Restarted during LTE	0	1	0	0	0	0	3	1	1	1	1	3	4	4
Total temporary interruptions (*n*) during study^c^	42	17	60	67	62	30	103	46	32	24	40	18	43	44
Patients with ≥ 1 interruption, *n* (%)	31 (14.8)	15 (9.4)	48 (22.3)	54 (11.1)	50 (10.3)	28 (8.5)	75 (15.4)	40 (12.1)	29 (12.7)	21 (9.2)	34 (15.0)	15 (8.5)	31 (17.8)	32 (18.1)
Number of interruptions per interrupted patient, mean (SD)	1.4 (0.6)	1.1 (0.4)	1.3 (0.4)	1.2 (0.5)	1.2 (0.4)	1.1 (0.3)	1.4 (0.6)	1.2 (0.4)	1.1 (0.4)	1.1 (0.4)	1.2 (0.4)	1.2 (0.6)	1.4 (0.6)	1.4 (0.7)
Time from first dose to first interruption, mean (SD), days	120.7 (100.9)	134.4 (113.5)	146.6 (97.9)	68.9 (43.0)	70.6 (48.6)	73.2 (49.4)	126.9 (93.4)	112.3 (74.0)	53.1 (40.1)	41.0 (39.3)	53.6 (37.7)	64.4 (39.1)	63.1 (46.1)	59.2 (43.1)
Duration of individual interruptions, mean (SD), days	16.3 (16.7)	15.0 (14.9)	17.5 (16.3)	11.7 (13.2)	11.4 (9.4)	19.4 (24.6)	15.1 (15.7)	23.1 (29.1)	11.6 (10.2)	12.3 (12.6)	10.7 (9.8)	16.8 (10.0)	12.9 (19.2)	12.6 (9.5)
Reason for interruptions, *n* (%)														
Adverse event	36 (85.7)	14 (82.4)	53 (88.3)	53 (79.1)	57 (91.9)	28 (93.3)	95 (92.2)	43 (93.5)	26 (81.3)	19 (79.2)	32 (80.0)	15 (83.3)	36 (83.7)	38 (86.4)
AE reported as an abnormal lab result^d^	9 (25.0)	0	9 (17.0)	6 (11.3)	9 (15.8)	7 (25.0)	–	–	2 (7.7)	1 (5.3)	0	1 (6.7)	1 (2.8)	1 (2.6)
Abnormal laboratory result	6 (14.3)	3 (17.6)	6 (10.0)	11 (16.4)	3 (4.8)	0	6 (5.8)	0	4 (12.5)	5 (20.8)	6 (15.0)	2 (11.1)	4 (9.3)	4 (9.1)
Investigator decision	0	0	1 (1.7)	3 (4.5)	2 (3.2)	2 (6.7)	2 (1.9)	3 (6.5)	2 (6.3)	0	2 (5.0)	1 (5.6)	3 (7.0)	1 (2.3)

Interruptions were based on daily tablet baricitinib study drug, including in non-baricitinib groups, which represent interruptions of the matching placebo for baricitinib. Temporary interruption is defined as a temporary withholding of study drug that is followed by resumption of study drug during the study

^a^Data up to rescue (all studies) or switch from PBO (RA-BEAM)

^b^No 0–52 week data for patients randomized to PBO because they were switched to baricitinib after week 24

^c^Interruption did not lead to permanent discontinuation and was therefore, by definition, considered a temporary interruption

^d^Percent is calculated with the number of adverse events as the denominator

*ADA* adalimumab, *AE* adverse events, *BARI* baricitinib, *LTE* long-term extension, *MTX* methotrexate, *PBO* placebo, *SD* standard deviation

The most common reason selected in the electronic case report forms by the investigators for temporary interruption was AE (Table 1), and the interruptions generally lasted 2 weeks or less (Fig. 1). Overall, a small proportion of interruptions (10.1%) where the reason chosen was AE were further reported by the investigator with an AE term related to an abnormal laboratory value and were thus not included in the overall count of the reason “abnormal laboratory result” but were counted in the reason “adverse event” (Table 1). There were no interruptions due to suspected pregnancies and few due to investigator decision. In all baricitinib-treated patients at any time including subsequent rescue, the most common AE leading to the interruption was infection and most of these were respiratory infections (Table 2) that were non-serious, and mild or moderate in severity.

**Table 2:** Detailed summary of reasons for interruptions of baricitinib in the phase 3 studies, with data from all patients receiving baricitinib at any time, including any subsequent rescue

	RA-BEGIN (weeks 0–52)	RA-BEAM (weeks 0–52)	RA-BUILD (weeks 0–24)	RA-BEACON (weeks 0–24)
	All baricitinib exposures (*N* = 400)	All baricitinib exposures (*N* = 972)	All baricitinib exposures (*N* = 511)	All baricitinib exposures (*N* = 407)
Total number of interruptions^a^	79	172	69	91
Reason for dose interruption^b^				
Adverse event	69	159	55	78
Infections (% total AEs)	39 (56.5)	93 (58.5)	36 (65.5)	55 (70.5)
Respiratory infections, *n* (% of infections)	26 (66.7)	60 (64.5)	18 (50.0)	38 (69.1)
Investigations (% of total AEs)^c^	6 (8.7)	3 (1.9)	1 (1.8)	1 (1.3)
Abnormal laboratory result^d^	9	8	12	8
Hepatic	5	1	3	1
eGFR/renal	1	1	2	2
Hemoglobin/hematocrit	3	2	0	0
Lymphocytes	0	2	1	2
Neutrophils	0	2	3	0
Creatine phosphokinase	0	0	2	2
Enrollment issue	0	0	1	1
Eosinophils	1	0	0	0
Myelocytes	0	0	1	0
Platelets	1	0	0	0

Patients who received any baricitinib dose includes patients who switched from placebo, adalimumab, or methotrexate to baricitinib, in addition to patients randomized to any baricitinib dose. Thus, these groups will be larger than the individual baricitinib treatment groups from each study added together

^a^Temporary interruption is defined as a temporary withholding of study drug that is followed by resumption of study drug during the study; total count includes interruptions occurring while assigned to any dose of baricitinib, either by initial randomization or after rescue from other treatments

^b^Reasons for interruption were examined for baricitinib-treated patients only, including patients randomized to placebo or active-control who were rescued or switched to baricitinib; Interruptions are based on daily tablet baricitinib study drug

^c^Investigations included clinical laboratory tests (including biopsies), radiologic tests, physical examination parameters, or physiologic tests (e.g., pulmonary function test); these included only investigation procedures and qualitative results and not conditions

^d^Reasons for an individual interruption could be associated with multiple abnormal laboratory results

AE, adverse event; eGFR, estimated glomerular filtration rate

### Impact of interruptions on efficacy

#### Signs and symptoms

**Fig. 2:**
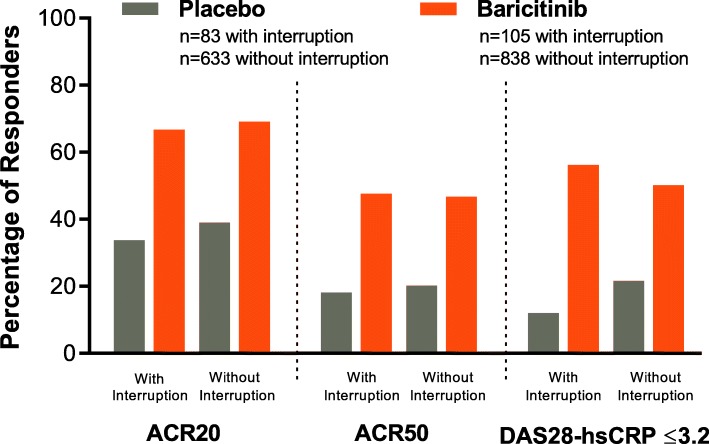
Percentage of 24-week responders among csDMARD/MTX-IR patients with/without interruption during the first 24 weeks. ACR20/50, 20%/50% improvement in American College of Rheumatology criteria; csDMARD, conventional synthetic disease-modifying antirheumatic drug; DAS28-hsCRP, Disease Activity Score based on a 28-joint count and high-sensitivity C-reactive protein; IR, inadequate responder; MTX, methotrexate

**Fig. 3:**
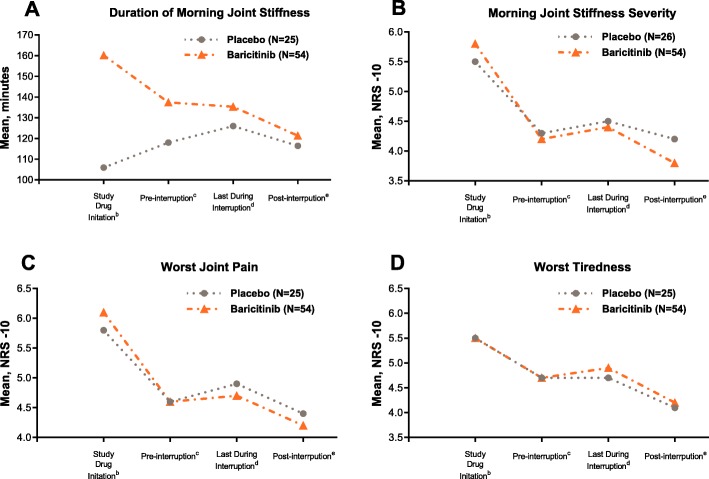
Time profile of daily diary scores among csDMARD/MTX-IR patients who were retreated following interruptions^a^. Data presented are combined from RA-BEAM and RA-BUILD for duration of morning joint stiffness (**a**) morning joint stiffness severity (**b**), worst joint pain (**c**), and worst tiredness (**d**); electronic diary data were gathered daily from week 0 to 12. ^a^Excludes interruptions without at least 3 diary entries during interruption. ^b^Average of values obtained within the first 3 days following randomization. ^c^Average of up to 3 most recent values obtained in the 7 days prior to interruption. ^d^Average of 3 most recent values in the last 7 days of the interruption. ^e^Average of last 3 available values obtained following interruption and prior to any subsequent interruption or week 12 study visit. *N*, number of interruptions with complete time profile; NRS, numeric rating scale

Patients who were csDMARD-IR (in RA-BEAM and RA-BUILD) and experienced interruptions had similar overall treatment responses as patients who never interrupted, as measured by ACR20, ACR50, and DAS28-CRP ≤ 3.2 at week 24 (Fig. 2). In these same studies in baricitinib-treated patients, daily diary measures of severity of MJS, worst joint pain, and worst tiredness showed modest increases during interruption (Fig. 3). There was no increase in duration of MJS. A similar pattern was observed in patients treated with placebo except for worst tiredness, which did not change during the interruption. Importantly, after resumption of study drug, responses measured with the daily diary returned to pre-interruption levels or better (Fig. 3). Patients with interruptions who were randomized to baricitinib (having an actual change to their treatment) had similar profiles of changes during and following interruption as patients randomized to placebo (having no actual change to their treatment).

#### Time to loss of disease control/increase in symptoms

Based on the response categories for the increase of symptoms during the interruption, 38 to 46% of patients reported no increases in their PRO symptoms from the daily diaries in RA-BEAM and RA-BUILD (Additional file 2: Table S2). The majority of baricitinib-treated patients (> 59% for MJS duration and 86 to 90% for MJS severity, worst tiredness, and worst joint pain) reported at most minimal changes (increase of ≤ 30 min for MJS duration, increase of ≤ 2 units for other PROs) in their daily PRO symptoms during the interruption. Approximately 6 to 9% of baricitinib-treated patients (except for 25% of patients for MJS duration) reported transitory larger increases in their symptoms (≥ 31 min for MJS duration, ≥ 3 units for other PROs) that became at most minimal increases at the time of last observation. For MJS severity and worst pain and tiredness, few baricitinib-treated patients (3 to 9%) reported more than minimal increases (≥ 3 units) in their symptoms that remained increased at the last observation during the interruption; slightly more (16%) experienced an increase of > 60 min in MJS duration (Additional file 2: Table S2). Among this last set of patients, such increases often occurred within the first week of the interruption. Based on these summaries, the most frequent greater-than-minimal increase in symptoms during interruptions was an increase in duration of MJS, followed by an increase in the worst tiredness, with infrequent greater-than-minimal increases in worst joint pain and severity of MJS. These increases in symptoms while interrupting baricitinib were similar among patients who interrupted placebo.

### Impact of interruptions on safety

#### Temporary interruptions due to AEs and reoccurrence after resumption

Adverse events were reported as the reason for 361 temporary interruptions while patients were taking baricitinib (Table 2). Of these interruptions, the same AE reoccurred in 32 cases (9%), after a mean of 58.4 days between reinitiation of study drug and the start of the reoccurring AE (range: 1 to 198 days). Twelve (38%) of these reoccurrences of AEs led to another temporary interruption in study drug and 4 (13%) led to permanent discontinuation. The most common reoccurring AEs were infections (*n* = 20, 6%).

#### Temporary interruptions due to laboratory abnormalities and reoccurrence after resumption

Abnormal laboratory results were reported as the reason for 37 temporary interruptions while patients were taking baricitinib (Table 2). Of the patients reporting these interruptions, 4 had a reoccurrence of a laboratory abnormality that occurred 38, 40, 53, and 141 days after reinitiation of study drug. None of the 4 reoccurrences led to another temporary interruption or were associated with any AEs of interest. One patient had neutrophil count equal to 0.56 × 10^9^ cells/L, leading to permanent discontinuation from the study.

#### Tolerability

In patients who experienced interruption of baricitinib during these studies, the most frequent AEs that occurred during the first 4 weeks on treatment included upper respiratory tract infection (URTI) (4.7% of patients who experienced an interruption), headache (4.1%), blood creatine phosphokinase (CPK) increased (3.1%), diarrhea (2.8%), nausea (2.5%), and bronchitis (2.5%) (Table 3). The frequencies of these same AEs in the first 4 weeks after baricitinib reinitiation following interruption were 1.6% (URTI), 0.6% (headache), 0.3% (CPK increased), 0.3% (diarrhea), 0.6% (nausea), and 0.9% (bronchitis) (Table 3). There were no specific AEs by MedDRA preferred term with a rate of occurrence after reinitiation that exceeded the rate during initial treatment by > 1%.

**Table 3:** Tolerability of baricitinib before and after temporary interruption based on adverse event terms reported by ≥ 2% of patients

*n* (%)	First 4 weeks after initiating baricitinib (*N* = 318)	First 4 weeks after restarting baricitinib (*N* = 318)
Patients with ≥ 1 TEAE	160 (50.3)	80 (25.2)
Upper respiratory tract infection	15 (4.7)	5 (1.6)
Headache	13 (4.1)	2 (0.6)
Blood creatine phosphokinase increased	10 (3.1)	1 (0.3)
Diarrhea	9 (2.8)	1 (0.3)
Bronchitis	8 (2.5)	3 (0.9)
Nausea	8 (2.5)	2 (0.6)
Constipation	7 (2.2)	0
Gastroenteritis	7 (2.2)	0
Urinary tract infection	7 (2.2)	1 (0.3)

*TEAE* treatment-emergent adverse event

## Discussion

Treatment of patients with rheumatoid arthritis requires long-term therapy that, at times, may need to be temporarily stopped. Despite this common clinical practice, there are little data about the efficacy response during treatment interruptions or safety outcomes. We attempted to characterize the rates of interruptions and the reasons patients temporarily discontinued study drug and to examine the effect of those interruptions on efficacy and safety outcomes.

During the baricitinib phase 3 trials, interruptions were infrequent and generally of short duration (≤ 2 weeks). In RA-BEAM, the duration of the interruptions was shorter for baricitinib (11.4 to 15.1 days) versus adalimumab (19.4 to 23.1 days). While the interruptions being summarized in this analysis are those for the oral tablets (baricitinib and placebo), the longer duration of interruptions for adalimumab could be due to the fact that baricitinib is administered daily and orally while adalimumab is an injection administered every 2 weeks, and if the tablets were interrupted in conjunction with a reason related to adalimumab, assessment to resume study drug might have been evaluated at longer intervals. In most studies, the interruptions occurred within the first 2 months after the first dose of study drug. The exception was in RA-BEGIN, where patients were MTX-naïve upon study entry and the average time to the first interruption after study drug initiation was approximately 4 to 5 months.

Interruptions were associated with minor increases in select RA symptoms in both the placebo and baricitinib groups, which resolved following resumption of therapy. The similarity between the response to withdrawal of placebo and active drug and the similarity of the improvement after resumption of the respective therapies suggests a nocebo effect [11] which was, however, relatively slight. Of note, no significant loss of response was apparent during these interruptions presumably because of their short-term nature. Similar results were seen with tofacitinib in patients who temporarily discontinued tofacitinib for 14 to 30 days. These patients had similar efficacy responses before and after interruption of the drug [12]. These data suggest that the recurrence of symptoms takes much longer than might be implied by the short half-life of the JAK inhibitors.

Unlike the assessment of RA symptoms during the interruption, we did not specifically assess the time taken for abnormal laboratory values to return to normal. The presumption was made that the investigator would not resume treatment if the values had not normalized (or increased/decreased enough during the interruption to remove the initial concern that caused the interruption). Additionally, the protocols for the studies had specific stopping and starting criteria regarding laboratory abnormalities. Therefore, the average duration for the interruptions could be used as a surrogate for the time for laboratory abnormalities to return to normal.

Adverse events leading to interruption generally did not reoccur upon reinitiation of treatment and, in those that did, few led to another interruption or permanent discontinuation. Adverse events that were observed in the first month of treatment with baricitinib tended to be observed less frequently upon retreatment, indicating that tolerability effects are likely to be short-term in nature and not an impediment to successful treatment with baricitinib in the long-term or when restarting treatment.

## Conclusion

Consistent with its pharmacologic properties, temporary interruption of baricitinib during phase 3 studies was not followed by significant reactivation of disease and baricitinib was rarely interrupted again after an initial interruption. The present study should serve to provide useful information for clinicians, as temporary interruption of antirheumatic therapy is common in the care of patients with RA.

## Supplementary information

**Additional file 1: Table S1.** Key study design features including patient population. Bari, baricitinib; bDMARD, biologic disease-modifying antirheumatic drugs; csDMARD, conventional synthetic disease-modifying antirheumatic drugs; IR, inadequate responder; MTX, methotrexate; SC, sub-cutaneous; TNFi, tumor necrosis factor inhibitor.

**Additional file 2: Table S2.** Flow chart of interruptions summarized in efficacy analyses of RA-BEAM and RA-BUILD.

**Additional file 3: Table S3.** Nature and timing of increase in symptoms/disease activity during temporary interruptions of baricitinib or matching placebo tablets. NRS, numeric rating scale.

## Data Availability

Lilly provides access to all individual participant data collected during the trial, after anonymization, with the exception of pharmacokinetic or genetic data. Data are available to request 6 months after the indication studied has been approved in the US and EU and after primary publication acceptance, whichever is later. No expiration date of data requests is currently set once data are made available. Access is provided after a proposal has been approved by an independent review committee identified for this purpose and after receipt of a signed data sharing agreement. Data and documents, including the study protocol, statistical analysis plan, clinical study report, and blank or annotated case report forms, will be provided in a secure data sharing environment. For details on submitting a request, see the instructions provided at www.vivli.org.

## Abbreviations

ACR20: American College of Rheumatology 20% response rate
AE: Adverse event
CPK: Creatine phosphokinase
DAS28-CRP: Disease Activity Score using 28 joints based on C-reactive protein
IR: Inadequate responder
MedDRA: Medical Dictionary for Regulatory Activities
MJS: Morning joint stiffness
NRS: Numeric rating scale
PRO: Patient-reported outcomes
RA: Rheumatoid arthritis
DMARD: Disease-modifying antirheumatic drugs
JAK: Janus kinase
csDMARD: Conventional synthetic disease-modifying antirheumatic drugs
MTX: Methotrexate
QD: Once daily
QW: Every week
