# Correction to: Temporary interruption of baricitinib: characterization of interruptions and effect on clinical outcomes in patients with rheumatoid arthritis

**DOI:** 10.1186/s13075-020-02257-1

**Published:** 2020-07-02

**Authors:** Paul Emery, Yoshiya Tanaka, Tracy Cardillo, Douglas Schlichting, Terence Rooney, Scott Beattie, Cameron Helt, Josef S. Smolen

**Affiliations:** 1 Leeds Muscoloskeletal Biomedical Research Centre/Chapel Allerton Hospital, Chapeltown Rd, Leeds, LS7 4SA UK; 2 The First Department of Internal Medicine, School of Medicine, University of Occupational and Environmental Health, Kitakyushu, Japan; 3 Eli Lilly and Company, Indianapolis, IN USA; 4 Division of Rheumatology, Department of Medicine 3, Medical University of Vienna, Vienna, Austria

**Correction to: Arthritis Research & Therapy (2020) 22: 115**

**https://doi.org/10.1186/s13075-020-02199-8**

Following publication of the original article [1], the authors identified errors in Fig. 1. The legends in Fig. 1c and d refer to placebo, baricitinib 4-mg and adalimumab treatment groups; the legends should refer to placebo, baricitinib 2-mg, and baricitinib 4-mg and the reference to these two studies in the figure caption are reversed. Additionally, the total number of interruptions for baricitinib 4-mg in RA-BEAM (Fig. 1b) has been corrected from 82 to 62 and the title for RA-BEAM has been corrected from 0-52 weeks to 0-24 weeks; labels have been corrected for MTX (8–14 days) in RA-BEGIN from 28 to 29 and for placebo (15–21 days) in RA-BUILD from 17 to 16.

The corrected Fig. 1 is given below.

**Fig. 1:**
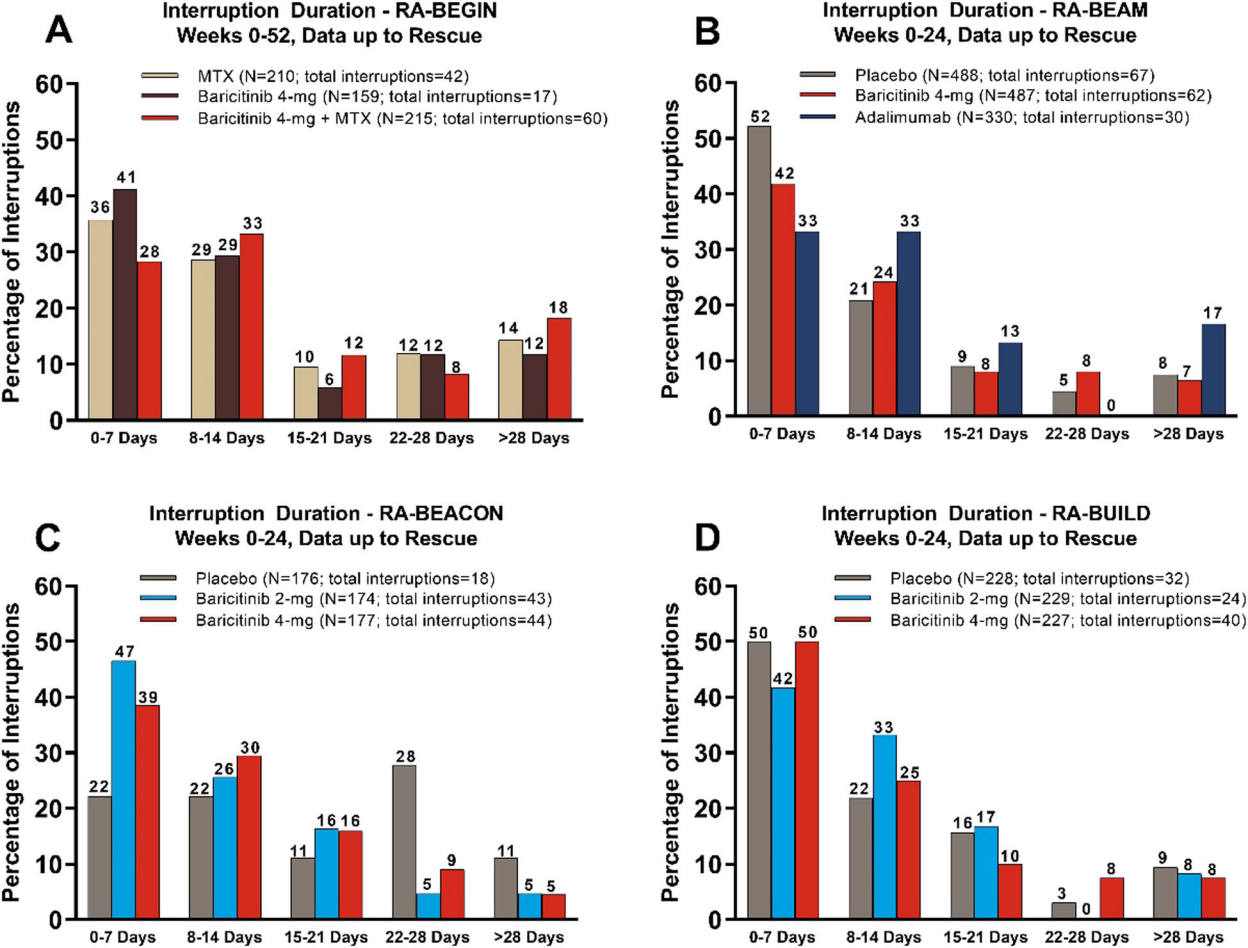
Duration of interruptions in the phase 3 studies RA-BEGIN (**a**), RA-BEAM (**b**), RA-BEACON (**c**), and RA-BUILD (**d**)^a,b^. ^a^Interruptions are based on daily tablet baricitinib study drug, including in non-baricitinib groups, which represent interruptions of the matching placebo for baricitinib. ^b^Temporary interruption is defined as a temporary withholding of study drug that is followed by resumption of study drug during the study. MTX, methotrexate
