# Supplementary material for: Temporary interruption of baricitinib: characterization of interruptions and effect on clinical outcomes in patients with rheumatoid arthritis
Source: Arthritis Res Ther. 2020 May 15;22:115. doi: 10.1186/s13075-020-02199-8 (PMC7227095; doi:10.1186/s13075-020-02199-8)
Supplement: Supplementary file 1 — Additional file 1: Table S1. Key study design features including patient population. Bari, baricitinib; bDMARD, biologic disease-modifying antirheumatic drugs; csDMARD, conventional synthetic disease-modifying antirheumatic drugs; IR, inadequate responder; MTX, methotrexate; SC, sub-cutaneous; TNFi, tumor necrosis factor inhibitor. [file 13075_2020_2199_MOESM1_ESM.docx]

Additional File 1: Table S1. Key study design features including patient population

|  | **Treatments** | **Prior RA treatments** | **Concomitant**  **therapies** | **Rescue (from week)** | **Rescue to** | **Study length (weeks)** |
| --- | --- | --- | --- | --- | --- | --- |
| **PHASE 3** |  |  |  |  |  |  |
| RA-BEGIN  NCT01711359 | MTX alone  Bari 4 mg alone  Bari 4 mg + MTX | DMARD-naïve | Not allowed | 24 | Open-label baricitinib 4-mg + MTX | 52: double-blind, active-control |
| RA-BEAM  NCT01710358 | Placebo  Bari 4 mg  Adalimumab | MTX-IR  bDMARD-naïve | Background MTX; optional csDMARDS | 16 | Open-label baricitinib 4-mg (SC injections stopped in rescued patients) | 24: double-blind, active- and placebo-control  28: double-blind, active-control |
| RA-BUILD  NCT01721057 | Placebo  Bari 4 mg  Bari 2 mg | csDMARD-IR  bDMARD-naïve | Optional csDMARDs;  no bDMARDs | 16 | Open-label baricitinib 4-mg | 24: double-blind, placebo-control |
| RA-BEACON  NCT01721044 | Placebo  Bari 4 mg  Bari 2 mg | TNFi-IR | Background  csDMARD | 16 | Open-label baricitinib 4-mg | 24: double-blind, placebo-control |

Bari, baricitinib; bDMARD, biologic disease-modifying antirheumatic drugs; csDMARD, conventional synthetic disease-modifying antirheumatic drugs; IR, inadequate responder; MTX, methotrexate; SC, sub-cutaneous; TNFi, tumor necrosis factor inhibitor
