# Supplementary material for: Temporary interruption of baricitinib: characterization of interruptions and effect on clinical outcomes in patients with rheumatoid arthritis
Source: Arthritis Res Ther. 2020 May 15;22:115. doi: 10.1186/s13075-020-02199-8 (PMC7227095; doi:10.1186/s13075-020-02199-8)
Supplement: Supplementary file 2 — Additional file 2: Table S2. Flow chart of interruptions summarized in efficacy analyses of RA-BEAM and RA-BUILD. [file 13075_2020_2199_MOESM2_ESM.docx]

Additional File 2: Table S2. Flow chart of interruptions summarized in efficacy analyses of RA-BEAM and RA-BUILD

|  | Placebo | Baricitinib |
| --- | --- | --- |
| Number of patients with interruptions of any duration during first 24 weeks of study | 83 | 105 |
| Number of patients with interruptions of any duration during first 24 weeks of study and with Week 24 response status for ACR20, ACR50, and DAS28-CRP (data presented in Figure 2: Percentage of responders at Week 24 among csDMARD/MTX-IR patients with or without interruption during the first 24 weeks (up to rescue) from RA-BEAM and RA-BUILD) | 83 | 105 |
| Number of patients with interruptions during first 12 weeks when daily patient-reported diaries were used (data presented in Additional File 3, Table S3: Nature and timing of increase in symptoms/disease activity during temporary interruptions of baricitinib or matching placebo tablets) | 52 | 69 |
| Number of patients with interruptions during first 12 weeks with patient-reported diary data that included at least 3 observations during the interruption and observations for all 4 time landmarks (baseline, pre-interruption, interruption, resumption); used for data in Figure 3 (Time profile of daily diary scores among csDMARD/MTX-IR patients who were retreated following treatment interruptions) | 26 | 54 |
