# Supplementary material for: Temporary interruption of baricitinib: characterization of interruptions and effect on clinical outcomes in patients with rheumatoid arthritis
Source: Arthritis Res Ther. 2020 May 15;22:115. doi: 10.1186/s13075-020-02199-8 (PMC7227095; doi:10.1186/s13075-020-02199-8)
Supplement: Supplementary file 3 — Additional file 3: Table S3. Nature and timing of increase in symptoms/disease activity during temporary interruptions of baricitinib or matching placebo tablets. NRS, numeric rating scale. [file 13075_2020_2199_MOESM3_ESM.docx]

Additional File 3. Table S3. Nature and timing of increase in symptoms/disease activity during temporary interruptions of baricitinib or matching placebo tablets

| **Diary Item** | **Change During Interruption** | **Placebo N=52** | **Baricitinib**  **N=69** |
| --- | --- | --- | --- |
| Morning Joint Stiffness Duration (minutes) | Never increased during interruption | 20 (39%) | 28 (41%) |
|  | Increased at most 30 minutes during interruption | 9 (17%) | 13 (19%) |
|  | Increased >30 minutes but resolved by end of interruption to ≤30 minutes increase | 12 (23%) | 17 (25%) |
|  | Increased and ended interruption >30 minutes with no increase >60 minutes in the first 14 days | 2 (4%) | 0 |
|  | Increased >60 minutes in >7 to ≤14 days and ended interruption >30 minutes | 0 | 0 |
|  | Increased >60 minutes in ≤7 days and ended interruption >30 minutes | 9 (17%) | 11 (16%) |
| Morning Joint Stiffness Severity  (0-10 NRS) | Never increased during interruption | 24 (46%) | 32 (46%) |
|  | Increased at most 2 units during interruption | 25 (48%) | 30 (44%) |
|  | Increased ≥3 units but resolved by end of interruption to ≤2 unit increase | 2 (4%) | 5 (7%) |
|  | Increased and ended interruption ≥3 units in >14 days | 0 | 1 (1%) |
|  | Increased and ended interruption ≥3 units in >7 to ≤14 days | 0 | 0 |
|  | Increased and ended interruption ≥3 units in ≤7 days | 1 (2%) | 1 (1%) |
| Worst Tiredness  (0-10 NRS) | Never increased during interruption | 23 (44%) | 26 (38%) |
|  | Increased at most 2 units during interruption | 22 (42%) | 33 (48%) |
|  | Increased ≥3 units but resolved by end of interruption to ≤2 unit increase | 6 (12%) | 4 (6%) |
|  | Increased and ended interruption ≥3 units in >14 days | 0 | 2 (3%) |
|  | Increased and ended interruption ≥3 units in >7 to ≤14 days | 0 | 0 |
|  | Increased and ended interruption ≥3 units in ≤7 days | 1 (2%) | 4 (6%) |
| Worst Joint Pain  (0-10 NRS) | Never increased during interruption | 24 (46%) | 27 (39%) |
|  | Increased at most 2 units during interruption | 19 (37%) | 34 (49%) |
|  | Increased ≥3 units but resolved by end of interruption to ≤2 unit increase | 6 (12%) | 6 (9%) |
|  | Increased and ended interruption ≥3 units in >14 days | 0 | 0 |
|  | Increased and ended interruption ≥3 units in >7 to ≤14 days | 1 (2%) | 0 |
|  | Increased and ended interruption ≥3 units in ≤7 days | 2 (4%) | 2 (3%) |

NRS, numeric rating scale
